# Early stages of divergence: phylogeography, climate modeling, and morphological differentiation in the South American lizard *Liolaemus petrophilus* (Squamata: Liolaemidae)

**DOI:** 10.1002/ece3.78

**Published:** 2012-04

**Authors:** Frank M Fontanella, Natalia Feltrin, Luciano J Avila, Jack W Sites, Mariana Morando

**Affiliations:** 1 Department of Biology and Chemistry, Morehead State UniversityMorehead, Kentucky; 2 CENPAT-CONICETBoulevard Almirante Brown 2915, Puerto Madryn, Chubut, Argentina; 3 Department of Biology and Bean Life Science Museum, Brigham Young UniversityProvo, Utah

**Keywords:** Ecological niche modeling, lizards, morphology, Patagonia, phylogeography, Pleistocene

## Abstract

This study examines the phylogeographic structure within the Patagonian lizard *Liolaemus petrophilus* and tests for patterns of between-clade morphological divergence and sexual dimorphism, as well as demographic and niche changes associated with Pleistocene climate changes. We inferred intraspecific relationships, tested hypotheses for historical patterns of population expansion, and incorporated ecological niche modeling (ENM) with standard morphological and geometric morphometric analyses to examine between-clade divergence as indirect evidence for adaptation to different niches. The two inferred haploclades diverged during the early Pleistocene with the Southern clade depicting the genetic signature of a recent population increase associated with expanding niche envelope, whereas the Northern clade shows stable populations in a shrinking niche envelope. The combination of molecular evidence for postisolation demographic change and ENM, suggest that the two haploclades have responded differently to Pleistocene climatic events.

## Introduction

Climatic fluctuations throughout the Quaternary have been widely recognized as one of the main natural historical processes influencing the genetic diversity of natural populations of the temperate northern hemisphere (Hewitt 1996, 2004). During the Pleistocene, the periodic expansions and contractions of glacial ice sheets resulted in latitudinal and altitudinal shifts in species’ ranges (Dynesius and Jansson 2000). For regions that experienced extensive cycles of glacial advance and retreat, such as Europe and North America, distinct genetic patterns can be inferred for populations persisting in or outside of ice age refugia (Soltis et al. 1997). Furthermore, climate changes and subsequent range shifts through the last 2.5 million years also affected the genetic composition of extant populations from unglaciated areas (Fontanella et al. 2008). Both phylogeographic and paleoecologic studies have shown that in heavily glaciated regions, populations expanded from southern refugia following the last glacial maximum (LGM) (Waltari et al. 2007; Fontanella et al. 2008). The geographically structured genetic diversity at southern latitudes can be attributed to long-term stable populations and/or the admixture of divergent lineages originating from separate refugia (Petit et al. 2003), whereas northern populations typically show decreased diversity attributed to rapid population expansion (Hewitt 1996, 1999; but see Rowe et al. 2004).

Recently, these predictions have been strengthened by the development of paleoclimate layers and the incorporation of ecological niche modeling (ENM) (Waltari et al. 2007). Under the assumption that a species’ climatic niche remains constant over time, these predictions can then be projected onto past or future climate layers to examine the effects of climatic changes on a species distribution. Ecological modeling has been used to determine the present-day distribution of species (Peterson 2001; Hijmans and Graham 2006; Raxworthy et al. 2009), infer refugial areas during the LGM (Waltari et al. 2007; Jezkova et al. 2009), and predict future distributions under varying climatic models (Martinez-Meyer 2005; Loarie et al. 2009). However, these methods only identify areas that are ecologically suitable, not areas that are necessarily inhabited by the species (Kambhampati and Peterson 2007). For instance, habitat specialization can affect a variety of traits that are important to a species niche including behavior (Moermond 1979), dispersal (Rice 1987), and morphology (Van Buskirk and Arioli 2005), which would not be incorporated into ENM models.

**Figure 1:**
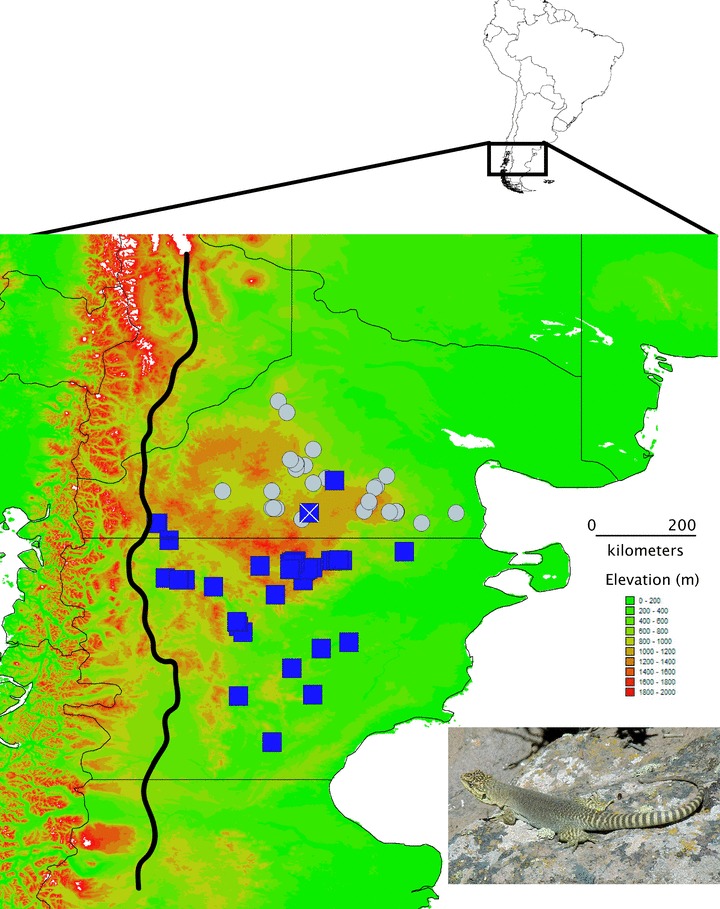
Distribution of *L. petrophilus* haplotypes around the Somuncurá Plateau inferred from the Bayesian analysis; symbols correspond to those in Figure 2. The white “X” inside the box represents the only location where haplotypes from the different clades were found syntopically. Bold irregular vertical line on the left represents the approximate eastern-most boundary for the last glacial maximum (LGM). Photograph of an adult *L. petrophilus* lizard. Photo credit: Natalia Feltrin.

Although there has been a recent increase of phylogeographic studies in South America (Graham et al. 2005; Ruzzante et al. 2008; Carnaval et al. 2009; Cosacov et al. 2010; Breitman et al. 2011; Nuñez et al. 2011; Sérsic et al. 2011), our understanding regarding the effects of climate change on the demographic history of South American species is in its infancy. From a temperate region perspective, only the southernmost portions of South America and New Zealand are comparable geographically with the glaciated regions of the northern hemisphere, and Patagonia has been identified as a global “blank spot” and thus a high priority for phylogeographic research (Beheregaray 2008). Throughout Patagonia, glaciers were confined to the north–south axis of the Andean Cordillera along much of their latitudinal range (Fig. 1; Sugden et al. 2005). Phylogeographic studies have identified southern refugia west of the Andes for some flowering plants (Muellner et al. 2005), freshwater fishes (Ruzzante et al. 2008), and frogs (Nuñez et al. 2011). However, the distributions of the focal species in these studies are in close proximity to the “Greatest Patagonian Glaciation,” which describes the maximum glacial extent during the Early Pleistocene between 1.2 and 0.7 million years ago (mya) (Kodama et al. 1986). The plateaus and low plains east of the Andes remained devoid of ice sheets, especially the Argentina Steppe (Cei 1969; Rabassa 2008). Thus, glacial refugia in this region may have been available for Patagonian species throughout their entire ranges. Further, extensive land areas east of the Argentinean coastline would have also been available as potential refugia during the LGM, due to the large shallow continental shelf that would have been exposed (Gutierrez and Martinez 2008). Thus, eastern Patagonia includes an historical geographic dimension without analogs of equal scale in the northern hemisphere or New Zealand.

In this study, we investigate the demographic history in two recently diverged haploclades of a central Patagonian lizard, *Liolaemus petrophilus* (Fig. 1), address morphological divergence between and within lineages and test for changes to demographic patterns and ecological niche envelopes through time. This medium sized lizard (65- to 112-mm snout vent length (SVL)) ranges across the Sub-Andean or Patagonian steppe environments along the eastern slope of the central Argentinean Andes between 350 and 1500 m, where ecologically it is typically saxicolous (Donoso-Barros and Cei 1971; Cei and Avila 1998; Avila et al. 2006a). Previous molecular work based on limited sampling identified two well-supported lineages within *L. petrophilus* positioned north and south of the Somuncurá Plateau (Morando et al. 2003). This basaltic tableland is isolated on an extended plain in northeastern Argentinean Patagonia, and includes three altitude levels: the filter creeks floor (500–900 m), the lowland floor (900–1500 m), and the volcanic heights (1500–2000 m) (Menni and Gomez 1995). Due to its central position, it was neither affected by marine transgressions nor glaciations during the Pleistocene (Rabassa 2008), and has served as an isolating barrier to haploclades of several other groups of lizards (Avila et al. 2006b; Morando et al. 2007). We expand upon the previous work (Morando et al. 2003) with additional sampling to examine the effects of late Pleistocene climate changes on the phylogeographic and demographic history of *L. petrophilus*. Phylogenetic concordance across nuclear loci is expected to require more time than mtDNA and is therefore less likely to be informative for recent divergences (Avise 2000; Zink and Barrowclough 2008). In this study, we incorporate classic and geometric morphometric analyses, ENM, and extend the mtDNA geographic sampling of Morando et al. (2003) to test for patterns of between-clade divergence, changes in niche potential through time, demographic changes, and sexual dimorphism. We consider the use of mtDNA a valid “hypothesis generating” approach (Avila et al. 2006b; Morando et al. 2007; Galbreath et al. 2009) and the details of the historical demography of the female *L. petrophilus* revealed in this study provide new insights into how central Patagonian species may have responded to climate fluctuations of the last 20,000 years.

## Materials and Methods

### Sampling and DNA extraction

Sampling was designed to cover the full altitudinal and latitudinal range of this species (Fig. 1). Tissue samples were stored in 96% ethanol and deposited in the Centro Nacional Patagónico Herpetological collection (LJAMM-CNP, CENPAT-CONICET, Puerto Madryn, Argentina, Table S1). Total genomic DNA was extracted from liver/muscle tissue following the protocol of Fetzner (1999) and using Qiagen DNeasy kits (Qiagen, Inc., Valencia, CA). Additionally, two outgroup taxa *L. austromendocinus* and *L. capillitas* were used based on the molecular phylogeny of Avila et al. (2004).

### Mitochondrial DNA amplification and sequencing

A total of 281 samples from 86 unique localities were sequenced for the cytochrome b gene region (804 bp) using the light strand primers GluDGL and the heavy strand primer Cyt b 3 (Palumbi et al. 1996). This region of cytochrome b has been shown to be informative at shallow levels of divergence in several other groups of *Liolaemus* (Morando et al. 2004, 2007; Avila et al. 2006b; Victoriano et al. 2008). For internal sequencing, we used the Cyt b 2 (Palumbi et al. 1996) and F1 (Whiting et al. 2003) primers.

Double-stranded PCR amplified products were purified using a MultiScreen PCR (mu) 96 (Millipore Corp., Billerica, MA) and directly sequenced using the BigDye Terminator v 3.1 Cycle Sequencing Ready Reaction (Applied Biosystems, Foster City, CA). Excess of Dye Terminator was removed with MultiScreen HV (Millipore Corp.), and sequences were fractionated by polyacrylamide gel electrophoresis on an ABI3730xl DNA Analyzer DNA sequencer (PE Applied Biosystems, Foster City, CA) at the DNA Sequencing Center at BYU. Sequences were deposited in GenBank under accession numbers JN846932–JN847213.

## Amplification and Sequencing of Nuclear Anonymous Locus LPB5C

A total of 20 specimens representative of each mtDNA haploclade were sequenced for the LPB5Cgene fragment. A 500-bp fragment of LPB5C was PCR amplified using the primers (F-CCATGGAACTCACTGGGATT, R-GATCAGTTGGCCCAGTTTTT), and a three-step amplification procedure. Reactions were performed in the following conditions: initial denaturation at 95°C for 90 sec, followed by 10 cycles of denaturation at 95°C for 35 sec, annealing at 63°C (decreasing by 0.5 degrees per cycle) for 35 sec, extension at 72°C for 60 sec; step two used an annealing temperature of 58°C for 10 cycles, and step three used an annealing temperature of 52°C for 10 cycles. Amplified products were sequenced using the amplification primers, and sequences were deposited in GenBank under accession numbers JN859142–JN859181.

## Phylogenetic Reconstruction and Dating of mtDNA Divergence

Sequences were edited and aligned using Sequencher (Gene codes, 2000, Ann Arbor, MI). No stop codons or indels were present in the alignments. Haplotypes were merged using the program Collapse version 1.2 (Posada 2006), and all trees rooted to *L. capillitas* and *L. austromendocinus* (AY367815) as outgroups (Avila et al. 2004).

Phylogenetic trees were constructed from distinct haplotypes using both maximum parsimony (MP) and Bayesian inference, in order to check for consistency in the results using algorithms based on different assumptions of molecular evolution. MP analysis were conducted in PAUP* 4.0b (Swofford 2002) using a heuristic search method with equally weighted characters, 1000 random addition–sequence replicates and the tree–bisection–reconnection (TBR) branch–swapping algorithm. Support for internal nodes was assessed using nonparametric bootstrapping (BS) (Felsenstein 1985) with 1000 pseudoreplicates and 100 random sequence–addition replicates.

We used BEAST v. 1.5.4 (Drummond and Rambaut 2007) to estimate the phylogeny and time since divergence within *L. petrophilus.* To insure an appropriate clock model and to test for deviation from a constant rate of molecular evolution (i.e., a “strict” molecular clock), we conducted a likelihood ratio test (LRT) implemented in the program HYPHY (Pond et al. 2005). Because a fossil calibration for *L. petrophilus* is currently lacking, we calibrated the tree by fitting a lognormal distribution around a sequence divergence rate of 1.6% per million years with a standard deviation of 0.12, and a relaxed uncorrelated lognormal clock model (see Results). The distribution of mutation rates encompassed the three substitution rates used in previous studies of *Liolaemus* (Morando et al. 2003) and is derived from previous studies using cytochrome b from other squamate reptiles (Giannasi 1997; Zamudio and Greene 1997; Malhotra and Thorpe 2000). Because we were working with intraspecific data, we used a constant size tree prior, as this model is more appropriate for these type of data (Ho 2007). Analyses were run for 50 million generations under the HKY +Γ model determined from jModeltest (Posada 2008) and sampled every 1000th generation. Results were visualized with TRACER v. 1.5 (Rambaut and Drummond 2005) following a preburnin of 15%. We fully recognize that this dating approach is far from ideal (Graur and Martin 2004), but a beginning exploration of recent phylogeographic histories of the biota of Patagonia in our view justifies provisional dating of this first split north and south of a major landscape feature, the Somuncurá Plateau (see also Breitman et al. 2011).

### Historical demography

Coalescent-based Bayesian skyline plots (Drummond et al. 2005) were generated for each clade with BEAST v 1.5.4 (Drummond and Rambaut 2007) to depict the change in female effective population size (N*_fe_*) from the time of the most recent common ancestor (TMRCA). We used the full dataset and performed five Markov Chain Monte Carlo (MCMC) runs for 20 million generations, sampling genealogy and population size parameters every 1000 generations and discarding the first 15% as burn-in. The HKY +Γ model of nucleotide substitution inferred from jModelTest was used to allow the sample space of the parameters to be explored. Default settings for the Bayesian priors were used. Demographic history through time was reconstructed using the software TRACER v. 1.3 (Rambaut and Drummond 2005).

### Estimation of climatic niche and distributional changes

The realized environmental niche of a species can be estimated from presence-only data with high precision by extracting niche dimensions from spatial information on the distribution of environmental parameters (Nix 1986). We used the maximum entropy model implemented in the program MAXENT v 3.3.1 (Phillips et al. 2006) to predict where the lineages of *L. petrophilus* are most likely to occur under current climatic conditions. MAXENT generates ecological niche models (ENM) using presence-only records, contrasting them with pseudoabsence data sampled from the remainder of the study area. We chose this approach because of its overall better performance with presence-only data and under conditions of small sample size (Elith et al. 2006). Given the limited morphological difference between lineages and thus the high level of uncertainty of assigning museum samples to the appropriate lineage, the contemporary ENMs were developed from occurrence points used in this study (*N*= 282; Table S1).

For the contemporary niche predictions, we used the 19 bioclimatic variables from the WorldClim data set (version 1.4) with a resolution of 2.5 min (Hijmans et al. 2005). Layers were trimmed to the areas surrounding each lineage and then projected over a larger region that included 38°35′ to 48°00′ and 63°20′ and 76°00′ (Anderson and Raza 2010). Bioclimatic variables were derived from monthly temperature and precipitation layers and represent biologically meaningful properties of climate variation (Hijmans et al. 2005; Waltari et al. 2007). For the LGM climate, the layers derived from the general circulation simulations using the Community Climate System Model (CCSM3) were used. The future layers (2100) were derived from the CCM3 climate model (Govindasamy et al. 2003). All layers are available from the WorldClim (http://www.worldclim.org/).

The models were run with the default convergence threshold (10^–5^), with a 1000 iterations and 25% of localities for model training. The program selected both suitable regularization values and functions of environmental values automatically, based on considerations of sample size. Because the samples removed for model training can affect the overall predicted distribution, we generated 10 models for each lineage and averaged the results using the cross-validation option. MAXENT outputs a continuous probability value (logistic values), which is an indicator of relative suitability for the species. Model clamping was checked with the “fade by clamping” option available in Maxent v 3.3.1. To determine the threshold value for each projection, we used the minimum training value averaged over the 10 runs. Layers projected with a 2.5-m arc resolution result in pixels equivalent to an area of 5 km^2^. Using this value, the number of pixels predicted as suitable habitat was determined and converted into area.

### Morphological data

A total of 180 individuals (102 males; 78 females) of *L. petrophilus* were examined for geometric morphometric quantification of shape variation, and 168 adult samples (93 males; 75 females) for classic morphometric variation, from the Chubut and Río Negro provinces.

### Classic morphometric/meristic characters

In order to test for morphological differences between clades, we chose characters that are similar to those used in earlier studies (Etheridge 2001; Etheridge and Christie 2003). Eight characters were studied: (SVL) snout-vent length, (MHW) maximum head width, (MHT) maximum head depth, (ND) internares distance, (AGD) axilla-groin distance, (4HL) fourth hind limb digit lamellae, and (3FL) third forelimb digit lamellae. All continuous measurements were taken from the left side of the animal using a Mitutoyo dial caliper (±0.05), as were the lamellae counts (with a stereomicroscope).

We tested normality in all characters with the Shapiro–Wilks test with Bonferroni's modification (Shapiro and Francia 1972; Mahibbur and Govindarajulu 1997), and homogeneity of variance with Levene's test (Sokal and Rholf 1998). We log transformed measurements for front limb length (FLL) to correct for the lack of normality. To examine differences between the north and south clades and considering between-sex differences in each clade, we used a two factor multivariate analysis of variance (MANOVA) with interaction and posterior Hotelling's comparisons to test the differences found in the MANOVAs (Hotelling 1936; Pillai 1960; Johnson and Wichem 1998), using the Infostat software (Di Rienzo et al. 2008).

### Geometric morphometrics

Shape, as defined by Kendall (1977), is the geometric information that remains after location, orientation, and scale have been filtered out. Shape can be described by a series of landmarks, which are points of correspondence between different objects that match between and within populations (Bookstein 1991). These landmarks, also know as Type 1 landmarks, have both coordinates and a biological significance (Bookstein 1991).

Procrustes methods utilize least square statistics to superimpose a given structure (target), at its corresponding landmarks onto a reference structure (Bookstein 1991). In the General Procrustes Analysis (GPA), all specimens are aligned to their mean shape based on a reference configuration. The results of the generalized Procrustes superimposition are scatters of corresponding landmarks (Procrustes shape coordinates) around their means. Therefore, the shape of a GPA superimposed landmark configuration is defined by the entirety of its residual coordinates. During the scaling procedure of GPA, a centroid size (the square root of the summed squared distances between the mean of all landmark coordinates of a specimen and each of the landmarks) is obtained. This transformation removes the correlation between size and shape (Zelditch et al. 2004), thus isolating cases of allometry in which correlations between size and shape can be observed.

Because lizard skulls have yielded abundant information for both phylogenetic and functional studies (Etheridge and de Quieroz 1988; Herrel et al. 1999; Schwenk 2000), we focused on 12 Type I landmark skull characters (Fig. S1). Specimens were photographed using a Sony T5 digital camera under natural light, and the landmarks were digitized on the dorsal view using the program tpsDig v 1.21 (Rohlf 2003). The coordinates and homologous landmarks were aligned and superimposed using GPA and projected in the Euclidean plane. Principal component analyses (PCA) and observation of the deformation graphics were calculated with TpsRelw v. 1.21. Because the MANCOVA showed significant covariation between variables, Mahalanobis distances were calculated from the PCA scores to quantify the variation in shape between males and females within each lineage. We then used Goodall's *F*-test for significance of the PCA scores between sexes within both lineages.

## Results

### Mitochondrial DNA

The cytochrome b gene resolved 62 unique haplotypes for 282 individuals with 615 constant and 106 parsimony informative characters. Unweighted MP analyses produced 60 equally parsimonious trees of 249 steps. The Bayesian analyses recovered a well-resolved phylogeny with a ln–likelihood score of –2272.517 and parameter value of alpha = 0.244. Both analyses produced highly congruent estimates of phylogenetic relationships for the major clades, therefore only the Bayesian (BI) phylogram is presented with posterior probabilities and mean estimates of time since divergence.

**Figure 2:**
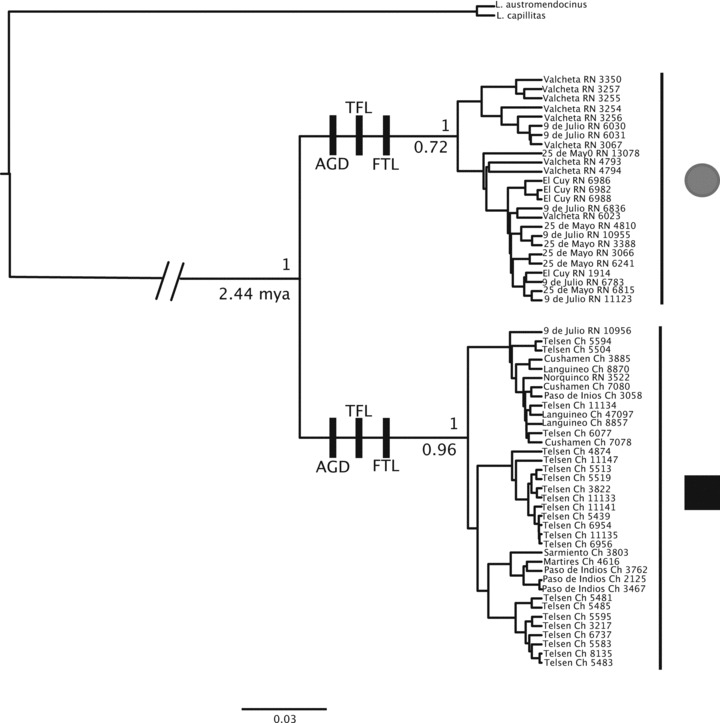
Bayesian phylogram of all unique *L. petrophilus* haplotypes; numbers above branches are posterior probabilities; numbers below branches are mean estimates of time in millions of years since divergence. Vertical bars represent the three morphological characters (axial groin distance [AGD], third finger lamellae of front limb [TFL], and fourth toe lamellae of hind limb [FTL]) that are significantly different (*P* < 0.05) between the north and south clades.

The BI tree recovers two well-supported clades within *L. petrophilus* (Fig. 2), with the distributions of haplotypes showing little overlap between clades and a north–south break associated with the Somuncurá Plateau. Three individuals representing two Southern clade haplotypes (H 33 and H 34) were found within the distribution of the Northern clade and in one instance haplotypes from both clades were found syntopically (Fig. 1). Within clades, phylogenetic structure was generally shallow and there was a complete lack of support for relationships among haplotypes within clades.

### Divergence dating and demographic analysis

**Figure 3:**
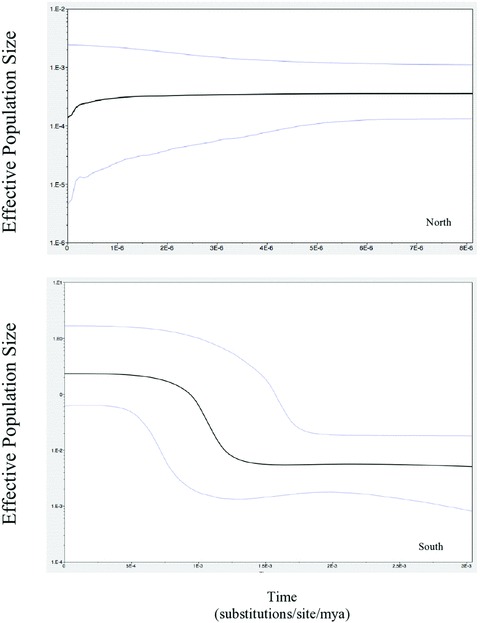
Bayesian skyline plots depicting the demographic history for each lineage of *L. petrophilus*. The solid line represents the median value for the log of the population size (log N_e_) and the dashed lines represent the upper and lower 95% credible intervals.

The assumption of a strict molecular clock was rejected by the LRT (*P* < 0.05), verifying the use of a relaxed clock. Results of the dating analysis suggest that the TMRCA for the two clades dates to the early Pleistocene with a mean estimate of 2.44 million years ago, while the TMRCA for each lineage occurred during the mid-Pleistocene (Fig. 2). The effective sample size (ESS) for each of the Bayesian skyline analyses was greater than 200, suggesting that the 20 million generations were sufficient to determine the demographic history for each lineage. Neither of the plots showed any evidence of population declines that would be typical of genetic bottlenecks or recent subdivisions (Fig. 3).

### Nuclear gene regions

For the 20 sequences analyzed for LPB5C gene fragment from across the species range, only six variable sites were found and these sites were not confined to a particular mtDNA clade. The lack of differentiating variation within these fragments may be due to the limited time since divergence between the clades, given that nuclear genes generally take much more time than mtDNA to coalesce, and are therefore less likely to be informative for recent divergences (Avise 2000; Zink and Barraclough 2008).

### Niche modeling

**Figure 4:**
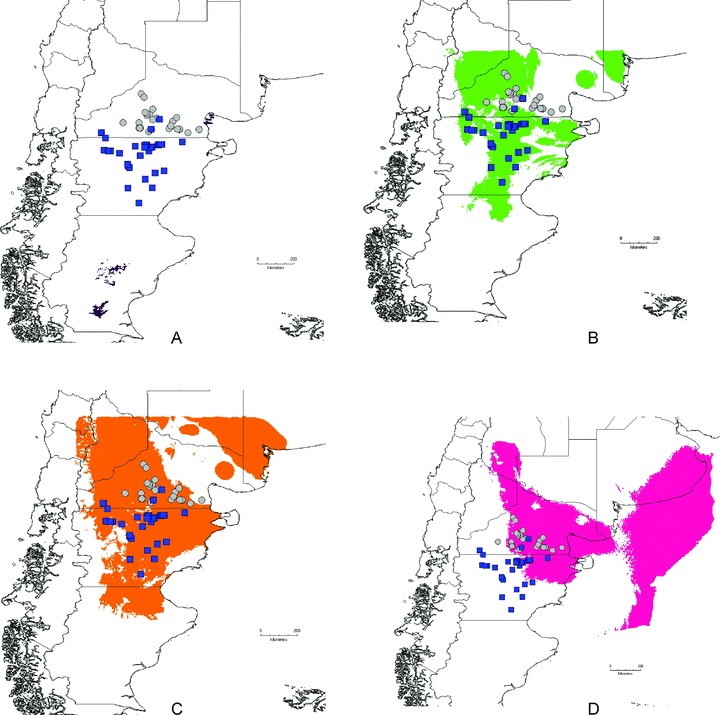

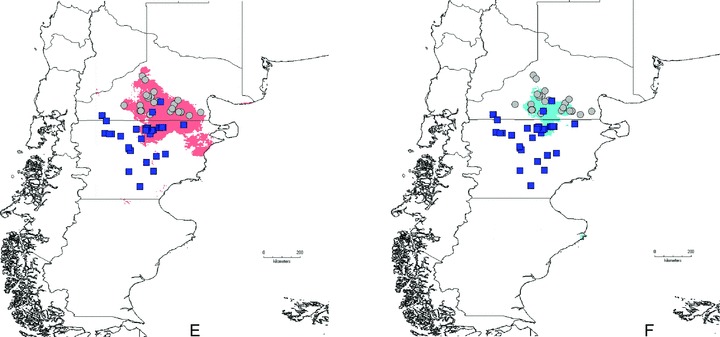
Ecological niche predictions for the Southern (A–C) and Northern (D–F) clades of *L. petrophilus* during the past (LGM) (A/D), present (B/E), and future (2100) (C/F) reconstructions, respectively. Predictions were inferred from CCSM3, WorldClim, and CCM3 climatic data sets, respectively. Symbols correspond to those in Figure 2. The present distribution of the two haplotypes is shown on all maps.

The present bioclimatic niche ranges for the haploclades of *L. petrophilus* are shown in Figure 4. Model validation was conducted by calculating the area under the curve (AUC), which reflects the model's ability to distinguish between presence records and random background points (Hanley and McNeil 1982). AUC values range from 0.5 for models without any predictive ability to 1.0 for models with perfect predictive ability. According to Swets (1988), AUC values >0.9 are considered to have “very good,” >0.8 “good,” and >0.7 “useful” discrimination abilities. The AUC scores were relatively high for both the Northern (0.92 ± 0.08) and Southern (0.87 ± 0.10) lineages. The predicted contemporary distribution for Northern clade closely matched the known range (Fig. 4E), whereas the same prediction for the Southern clade (Fig. 4B) showed a broader suitable range overlapping with the western portion of the Northern clade and extending to the northwest. Despite the overlap between predictions, the variables with the greatest contribution to the models differed for each lineage. For the Northern clade, three variables (BIO3: Isothermality, BIO10: mean temperature warmest quarter, and BIO9: mean temperature of driest quarter) accounted for 73% of the predicted range, whereas in the Southern clade three different variables (BIO13: precipitation of the wettest month, BIO14: precipitation of driest month, and BIO5: maximum temperature of the warmest month) accounted for 61% of the predicted range.

The LGM niche predictions depict striking contrasts for the two lineages. The Southern clade was isolated into small refugia south and possibly east of the present distribution, and has undergone a substantial increase in range (1043%; Fig. 4A and 4B). Conversely, the Northern clade likely had a more extensive LGM distribution, possibly extending far to the east of the present coastline on the exposed continental shelf, and has since experienced 86% decrease in overall range size from the LGM to the present (Fig. 4D and 4E).

The projection of the future suitable area of the Southern clade showed an increase in range size (88%) compared to contemporary models (Fig. 4B and 4C). The predictions for the Northern clade showed a continued decline in suitable habitat toward a distribution that would be 71% smaller than the contemporary range (Fig. 4E and 4F; Table 1).

**Table 1:** Area of predicted suitable habitat for the inferred lineages of *L. petrophilus* during the last glacial maximum (LGM) (∼21,000 ya), Present, and Future (2100).

Lineage	LGM area (km^2^)	Present area (km^2^)	Future area (km^2^)
Northern	37,445	5170	1470
Southern	1380	15,780	29,750

Area calculated as the total number of 2.5-km pixels predicted as suitable under a binary threshold and multiplied by 5 (2.5 km of arc = 5 km^2^).

## Morphological Data

### Classic morphometric/meristic analyses

Lizards smaller than 65-mm SVL were considered juveniles and removed from all analyses. An *F*-test revealed significant differences between lineages (*F*_8,157_= 9.01; Wilks’ Lambda = 0.70; *P* < 0.001), specifically for six continuous (SVL, MHW, MHT, ND, AGD, lnFLL) and two meristic (4HL and 3FL) characters (Table S2). Without considering differences between clades, sexual dimorphism was detected (*F*_8,157_= 13.15; Wilks’ Lambda = 0.61; *P* < 0.001), but we did not find a significant interaction between sex and clade (*F*_8,157_= 1.82; Wilks’ Lambda = 0.91; *P* < 0.056). However, for the posterior Hotelling's comparisons, all tests are significant only for the Northern clade (*P* < 0.01; Table 2); sexual dimorphism is present but not significant in the Southern clade. For the interclade analysis, we found six continuous (SVL, MHW, MHT, ND, AGD, lnFLL) and two meristic (4PFL and 3FFL) characters that differed significantly between lineages (*P*< 0.001).

**Table 2:** Posterior Hotelling's Comparisons (with Bonferroni's modification) that showed detail significant use of different sexes and different clades as factors for *L. petrophilus.*

	North clade— female	North clade— male	South clade— female	South clade— male
North clade—female		*P* < 0.01*	*P* < 0.01*	*P* < 0.01*
North clade—male	4.57		*P* < 0.01*	*P* < 0.01*
South clade—female	2.00	2.72		NS
South clade—male	5.77	2.24	1.28	

* , statistically significant; NS, not significant.

### Geometric morphometric analysis

**Figure 5:**
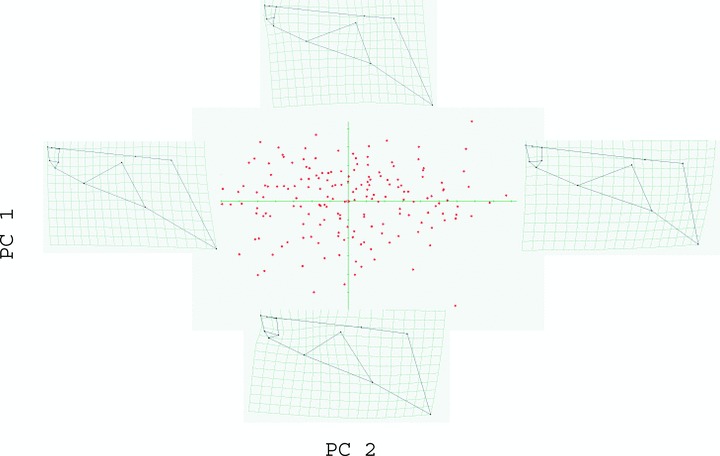
Relative warps (RW) for values of α= 0 for *L. petrophilus*; each dot represents the value for each individual for the first two axes, and in the deformation grids for the extreme shapes, the horizontal axis depicts snout shape change (variability explained = 40.13%), while the vertical axis holds this dimension and adds shape change to the orbital region (variability explained = 10.98%).

The first two PCA roots (relative warps) of shape variables of *L. petrophilus* show differences between north and south clades, and between males and females. The 12 landmarks account for 51.1% of the total variation among individuals (Fig. 5). The horizontal axis (first relative warp score) explains 40.13% of the variation, whereas the vertical axis (second relative warp score) explains 10.98% of the total shape variation in head morphology among individuals. Visualization of the warp grids shows that along the first principal component, the variation is mainly in the sagittal axis, in the anteroposterior sense. Along the second principal component, there is evidence of a shift in the relative position of the landmarks, the snout shape is maintained and the ocular orbit landmarks show modifications (Fig. 5). The results of the Goodall's *F*-test were not significant between sexes for either clade at the 0.05 level. However, the head shape between males and females of the Northern clade showed a greater, nearly significant, amount of divergence (Goodall's statistics = 1.41; *P*= 0.12) than between males and females of the Southern clade (Goodall's statistics = 0.95; *P*= 0.52). Overall and in accordance with the classic morphometric analysis, the Northern clade's head shape is more robust than that for the Southern clade, and head shape variation is greater between sexes in the Northern clade.

## Discussion

### Phylogeographic patterns

Overall, our results confirm and extend previous molecular work on *L. petrophilus* (Morando et al. 2003); two widespread haploclades are separated from each other on a north/south axis associated with the Somuncurá Plateau (Figs. 1 and 2), a pattern corroborating previous findings of other Patagonian lizards (*L. melanops; L. bibronii)* (Avila et al. 2004; Morando et al. 2007). Although a direct mutation rate is not available for *L. petrophilus,* the range of divergence estimates suggests that the split between phylogroups occurred during the early Pleistocene and therefore would have been subjected to climatic changes during the LGM.

The two phylogroups have also come into secondary contact at a single point along the north/northwest side of the Somuncurá Plateau, within which we identified one location that contained mtDNA from both Northern and Southern clades. The extent of hybridization occurring at this site is difficult to infer due to limited sample sizes, but the issue merits further study.

### Demographic history

During the Pleistocene, the periodic expansions and contractions of glacial ice sheets resulted in latitudinal and altitudinal shifts in species’ ranges (Dynesius and Jansson 2000). Phylogeographic studies have shown that in heavily glaciated northern hemisphere regions, populations at northern latitudes were pushed south and then experienced rapid northward expansions following glacial retreat, whereas southern populations in these regions remained relatively stable (Kozak et al. 2006; Fontanella et al. 2008). Unlike the northern hemisphere, little information exists on the effects of historical climate change on temperate South American species (but this is changing rapidly; Ruzzante et al. 2008; Cosacov et al. 2010; Breitman et al. 2011; Nuñez et al. 2011; Sérsic et al. 2011). Our demographic analyses depict contrasting patterns compared to the typical expansion–contraction models of the northern hemisphere. The Bayesian skyline plot for the Southern clade is consistent with rapid population expansion, whereas the Northern clade did not show any evidence of an historical rapid increase in population size (Fig. 3). Additionally, the slope of the skyline plot for the Northern clade was not significantly different from zero (assuming constant population size), suggesting relative demographic stability rather than the rapid population expansion typically associated with postglacial shifts typical of northern hemisphere taxa.

### Ecological niche modeling

Although the habitat suitability inferred from ENM results is not an absolute prediction of the true fundamental or realized niche of an organism, it should provide a reasonable proxy for testing hypotheses with respect to niche preferences, at least with regard to the major environmental conditions experienced by organisms (Kozak et al. 2008; Warren et al. 2008). Ecological niches will usually be conserved in the sense that descendent populations will inhabit similar geographical areas or ecological niches as their immediate ancestors (Wiens and Graham 2005; Losos 2008; Wiens 2008). Even though the contemporary ENM predictions for the Northern and Southern clades are not mutually exclusive, the separate LGM refugia suggest that the ecological requirements between sister lineages have not been broadly conserved to the degree that they share identical ENMs (Fig. 4). Indeed our findings that the ecological variables differed for each lineage is consistent with those of Rodriquez–Serrano et al. (2010), who showed that the thermal physiology of *Liolaemus* lizards is evolutionarily flexible and that this ecological plasticity has been partially responsible for the colonization of a wide array of thermal environments. This pattern suggests that species may evolve ecologically significant differences between recently diverged haploclades as natural selection acts on populations in ecologically heterogeneous habitats (Wiens 2004).

Consistent with previous studies examining the effects of climate change during the LGM (Waltari et al. 2007; Jezkova et al. 2009), our paleoclimate reconstructions predicted a smaller area of suitable habitat for the Southern clade (Fig. 4A and 4B) and possible LGM refugia outside the current predicted and known range (Fig. 4). However, extensive sampling has not recovered any *L. petrophilus* from within this region, a finding consistent with the hypothesis of niche conservatism in which species track their niche over time (assuming that a species’ climatic niche requirements remain constant). Since the LGM, the suitable habitat has not only undergone a geographic shift but also a substantial increase (1043%) in overall size, a result consistent with our findings of rapid postglacial expansion (assuming an expansion contraction model).

In contrast, the cooler and drier climatic conditions during the Pleistocene provided more favorable environmental conditions for the Northern clade. Backcasting onto the LGM layers predicted a substantially larger area that encompassed the present day distribution (Fig. 4D and 4E). As the climate warmed, the available climatic niche underwent a substantial decrease (86%) resulting in the smaller present day “refugium.” This decrease in available climate niche space is further reflected in the relatively stable demographic history inferred from the Bayesian skyline plot.

Because we found contrasting patterns of niche prediction associated with climate change from the LGM to present day (i.e., increasing global temperatures), we forecasted our predictions onto future layers to infer the possible effects of continued global warming. These climate layers are derived under an assumption of doubled atmospheric CO_2_ levels by the year 2100. For the Southern clade, the pattern of range expansion will continue to increase with increasing global temperatures (Fig. 4C), and expand northwards into the current distribution of the Northern clade, possibly occupying up to 88% more area than its current range (Table 1).

Under the same model of global warming, the predicted niche for the Northern clade will continue to decline (Fig. 4 F), decreasing by an estimated 71% (Table 1). This does not necessarily mean that these habitats will remain unoccupied. In the absence of *L. petrophilous*, these areas will possibly be colonized by ecologically similar species presently inhabiting adjacent areas. Indeed, this process may be currently underway, as evidenced by the position of Southern clade haplotypes within the range of the Northern clade, and the potential for further northern range expansion of the Southern clade with increasing climate temperatures.

The predicted range contraction of the Northern clade raises the issues of its possible extinction from portions of its former range, and the impact on the overall genetic variability of the species. This is illustrated by the potential loss of haplotypes occurring exclusively in populations located in the predicted unsuitable area under future models (Fig. 4E). The disappearance of the those populations and the overtaking of the Northern clade due to expansion of the Southern clade could influence the overall survival of the lineage as a whole due to its genetic impoverishment and compromised adaptive potential.

### Morphological evidence for divergence

Evolutionary biologists are faced with several challenges in understanding the role of climate change on populations. Rapid climate change is likely to impose strong selection pressures on traits that are important for fitness (Sinervo et al. 2010). Therefore, understanding microevolutionary responses to climate-mediated selection is an important factor in understanding the consequences of global climate change. Evidence for phenotypic responses to climate change includes advances in phenology (Parmesan and Yohe 2003), shifts in distributional ranges (Walther et al. 2005; Parmesan 2006), changes in population phenotypes such as body size (Vidal et al. 2005, 2006; Cruz et al. 2005, 2011; Millien et al. 2006) and extinctions (Sinervo et al. 2010). Our morphological analyses combined with the ENM provide a window into the possible cause of significant sexual size dimorphism (SSD) within the Northern clade. Sexual dimorphism in size and morphology is widespread in animals, a pattern that has been explained by three mechanisms: sexual selection, fecundity selection, and ecological causation (e.g., resource partitioning) (Gaulin and Sailer 1985; Shine 1989). In lizards, changes in prey choice are often correlated with changes in head morphology (Metzger and Herrel 2005; Brecko et al. 2008). The deformation grids show that changes in cranial morphology are associated with snout and mandible shape (Fig. 5), with the Northern clade having larger, more robust heads. Our ENM models depict a continuous loss of suitable habitat for the Northern clade, possibly resulting in an increased pressure on available resources. When resources are limited or patchy, resource partitioning may be selected for to reduce intraspecific competition. Our warp plot analyses show that changes to snout shape are greater between sexes in the Northern relative to the Southern clade, a pattern that has been linked to changes in prey choice (Metzger and Herrel 2005; Brecko et al. 2008). Differences between sexes in the Northern clade are significant at the *P* < 0.001 level, which may represent early stages of the evolution of sexual dimorphism, and a contrasting pattern within the Southern clade where there is no evidence for sexual dimorphism.

An alternative is that the morphological differences between Northern and Southern clades simply reflect adaptations to one or more niche axes in their respective distributions, independent of range shifts. While further research into the underlying cause(s) of SSD is needed, these combined data provide evidence for a different aspect of niche divergence between the *L. petrophilus* haploclades (for biotic attributes not included in the ENM), and generate testable hypotheses regarding the evolution of SSD in this species.

## Supporting information

**Figure S1.** The 12 Type I landmarks used in the morphometric analysis and variation between samples after removal of nonshape variation.

**Table S1.** Collecting localities and museum numbers for samples used in this study.

**Table S2.** Continuous and meristic characters used in theclassic morphometrics analysis of *L. petrophilus*.
