# Supplementary material for: Early stages of divergence: phylogeography, climate modeling, and morphological differentiation in the South American lizard Liolaemus petrophilus (Squamata: Liolaemidae)
Source: Ecol Evol. 2012 Apr;2(4):792–808. doi: 10.1002/ece3.78 (PMC3399201; doi:10.1002/ece3.78)

Supplementary Figure 1: The twelve Type I landmarks used in the morphometric analysis and variation between samples after removal of non-shape variation.


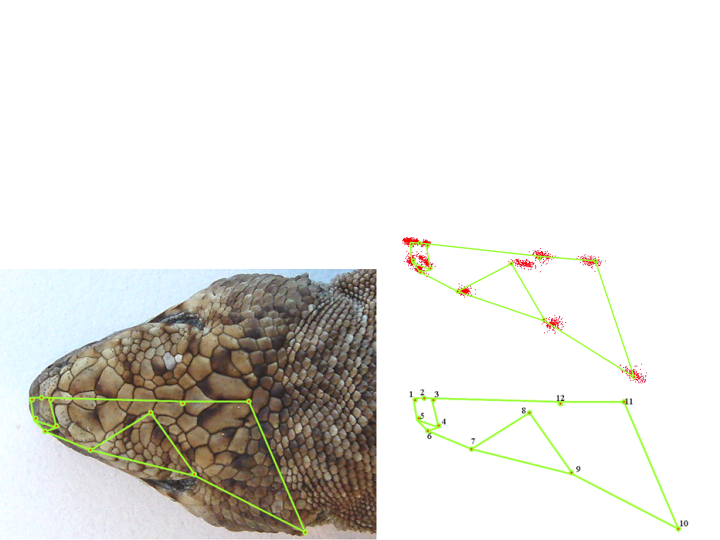

Supplement: Supplementary file 1 [file ece30002-0792-SD1.doc]
