# Supplementary material for: Early stages of divergence: phylogeography, climate modeling, and morphological differentiation in the South American lizard Liolaemus petrophilus (Squamata: Liolaemidae)
Source: Ecol Evol. 2012 Apr;2(4):792–808. doi: 10.1002/ece3.78 (PMC3399201; doi:10.1002/ece3.78)
Supplement: Supplementary file 2 [file ece30002-0792-SD2.doc]

| Colection number  Supplementary Table 1. Collecting localities and museum numbers for samples used in this study. Asterisks indicate samples for which genetic and morphometric data were generated. | Provincia | Latitude | Longitud3 | Cyt-b | LPB5C | MG | MC | MN |
| --- | --- | --- | --- | --- | --- | --- | --- | --- |
| 1612-LJAMM-CNP | Río Negro | 39º55'S | 68º20'W |  |  | * | * |  |
| 1613-LJAMM-CNP/FML 08574 | Río Negro | 39º55'S | 68º20'W |  |  |  | * |  |
| 1614-LJAMM-CNP/FML 08575 | Río Negro | 39º55'S | 68º20'W |  |  |  | * |  |
| 1652-LJAMM-CNP | Río Negro | 40º 17'S | 68º 27'W |  |  | * | * |  |
| 1761-LJAMM-CNP | Río Negro | 39º55'S | 68º20'W |  |  | * | * |  |
| 1762-LJAMM-CNP | Río Negro | 39º55'S | 68º20'W |  |  | * | * |  |
| 1763-LJAMM-CNP | Río Negro | 39º55'S | 68º20'W |  |  | * | * |  |
| 1764-LJAMM-CNP | Río Negro | 39º55'S | 68º20'W |  |  | * | * |  |
| 1765-LJAMM-CNP | Río Negro | 39º55'S | 68º20'W |  |  | * | * |  |
| 1842-LJAMM-CNP | Río Negro | 39º55'S | 68º20'W |  |  | * | * |  |
| 1843-LJAMM-CNP | Río Negro | 39º55'S | 68º20'W |  |  | * | * |  |
| 1844-LJAMM-CNP | Río Negro | 39º55'S | 68º20'W |  |  | * | * |  |
| 1845-LJAMM-CNP | Río Negro | 39º55'S | 68º20'W |  |  | * | * |  |
| 1846-LJAMM-CNP | Río Negro | 39º55'S | 68º20'W |  |  | * | * |  |
| 1847-LJAMM-CNP | Río Negro | 39º55'S | 68º20'W |  |  | * | * |  |
| 1848-LJAMM-CNP | Río Negro | 39º55'S | 68º20'W |  |  | * | * |  |
| 1914-LJAMM-CNP /BYU 47098 | Río Negro | 39º55'S | 68º20'W | * |  |  | * | * |
| 2125-LJAMM-CNP | Chubut | 43º49'S | 67º45'W | * |  |  | * | * |
| 2126-LJAMM-CNP/FML 13046 | Chubut | 43º49'S | 67º45'W | * |  | * |  | * |
| 2132-LJAMM-CNP | Río Negro | 41º45'S | 70º31'W | * | * | * | * | * |
| 2133-LJAMM-CNP | Río Negro | 41º45'S | 70º31'W | * | * | * | * | * |
| 2140-LJAMM-CNP | Río Negro | 40º42'S | 68º16'W | * |  | * | * | * |
| 2158-LJAMM-CNP | Río Negro | 40º42'S | 68º16'W | * |  |  |  | * |
| 2239-LJAMM-CNP /BYU 47096 | Río Negro | 40º42'S | 68º16'W | * |  |  | * | * |
| 2396-LJAMM-CNP | Río Negro | 39º55'S | 68º20'W | * |  | * | * | * |
| 2809-LJAMM-CNP/FML 13063 | Chubut | 42º48'S | 69º34'W | * |  |  | * | * |
| 2810-LJAMM-CNP /BYU 47097 | Chubut | 42º48'S | 69º34'W | * |  | * | * | * |
| 3058-LJAMM-CNP | Chubut | 43º27'S | 69º08'W | * |  | * | * | * |
| 3059-LJAMM-CNP/FML 13074 | Chubut | 43º27'S | 69º08'W | * |  | * | * | * |
| 3060-LJAMM-CNP | Río Negro | 41º30'S | 68º33'W | * |  | * | * | * |
| 3061-LJAMM-CNP | Río Negro | 41º30'S | 68º33'W | * |  | * | * | * |
| 3062-LJAMM-CNP/FML 13075 | Río Negro | 41º30'S | 68º33'W | * |  | * | * | * |
| 3063-LJAMM-CNP | Río Negro | 41º30'S | 68º33'W | * |  | * | * | * |
| 3064-LJAMM-CNP | Río Negro | 41º13'S | 69º24'W | * |  | * | * | * |
| 3066-LJAMM-CNP | Río Negro | 41º30'S | 68º33'W | * |  | * | * | * |
| 3067-LJAMM-CNP | Río Negro | 41º45'S | 66º04'W | * |  | * | * | * |
| 3068-LJAMM-CNP/FML 13076 | Río Negro | 41º45'S | 66º04'W | * |  | * | * | * |
| 3069-LJAMM-CNP | Río Negro | 41º45'S | 66º04'W | * |  | * | * | * |
| 3070-LJAMM-CNP | Río Negro | 41º45'S | 66º04'W | * |  | * | * | * |
| 3075-LJAMM-CNP/FML 13078 | Río Negro | 41º13'S | 69º25'W | * |  | * | * | * |
| 3076-LJAMM-CNP | Río Negro | 41º13'S | 69º24'W | * |  | * | * | * |
| 3077-LJAMM-CNP | Río Negro | 41º13'S | 69º24'W | * | * | * | * | * |
| 3078-LJAMM-CNP | Río Negro | 41º13'S | 69º24'W | * | * | * | * | * |
| 3079-LJAMM-CNP/FML 13079 | Chubut | 42º13'S | 66º22'W | * |  | * | * | * |
| 3080-LJAMM-CNP | Río Negro | 41º45'S | 69º24'W | * |  | * | * | * |
| 3084-LJAMM-CNP | Río Negro |  |  |  |  | * | * |  |
| 3085-LJAMM-CNP | Río Negro |  |  |  |  | * | * |  |
| 3086-LJAMM-CNP/FML 13081 | Río Negro |  |  |  |  |  | * |  |
| 3215-LJAMM-CNP | Chubut | 42º29'S | 67º53'W | * |  | * | * | * |
| 3216-LJAMM-CNP | Chubut | 42º29'S | 67º53'W | * |  | * | * | * |
| 3217-LJAMM-CNP | Chubut | 42º29'S | 67º53'W | * |  | * | * | * |
| 3218-LJAMM-CNP | Chubut | 42º21'S | 69º56'W | * | * | * | * | * |
| 3219-LJAMM-CNP | Chubut | 42º21'S | 69º56'W | * | * | * | * | * |
| 3220-LJAMM-CNP | Chubut | 42º21'S | 69º56'W | * | * | * | * | * |
| 3231-LJAMM-CNP | Río Negro | 41º33'S | 66º29'W | * |  | * | * | * |
| 3254-LJAMM-CNP | Río Negro | 41º34'S | 66º30'W | * |  | * | * | * |
| 3255-LJAMM-CNP | Río Negro | 41º34'S | 66º30'W | * | * | * | * | * |
| 3256-LJAMM-CNP | Río Negro | 41º34'S | 66º30'W | * |  | * | * | * |
| 3257-LJAMM-CNP | Río Negro | 41º34'S | 66º30'W | * |  | * | * | * |
| 3258-LJAMM-CNP | Río Negro | 41º34'S | 66º30'W | * |  | * |  | * |
| 3271-LJAMM-CNP | Río Negro | 41º33'S | 66º30'W | * |  | * | * | * |
| 3314-LJAMM-CNP | Río Negro | 41º33'S | 66º36'W | * |  | * | * | * |
| 3315-LJAMM-CNP | Río Negro | 41º33'S | 66º36'W | * |  | * | * | * |
| 3316-LJAMM-CNP | Río Negro | 41º33'S | 66º36'W | * |  | * | * | * |
| 3321-LJAMM-CNP | Río Negro | 41º25'S | 66º57'W | * |  | * | * | * |
| 3347-LJAMM-CNP | Río Negro | 41º10'S | 66º51'W | * |  | * | * | * |
| 3348-LJAMM-CNP | Río Negro | 41º08'S | 66º50'W | * |  | * | * | * |
| 3349-LJAMM-CNP | Río Negro | 41º08'S | 66º50'W | * |  | * | * | * |
| 3350-LJAMM-CNP | Río Negro | 41º08'S | 66º50'W | * |  | * | * | * |
| 3351-LJAMM-CNP | Río Negro | 41º08'S | 66º50'W | * |  | * | * | * |
| 3362-LJAMM-CNP | Río Negro | 40º47'S | 68º02'W | * |  | * | * | * |
| 3363-LJAMM-CNP | Río Negro | 40º47'S | 68º02'W | * |  | * | * | * |
| 3364-LJAMM-CNP | Río Negro | 40º47'S | 68º02'W | * |  | * | * | * |
| 3365-LJAMM-CNP | Río Negro | 40º47'S | 68º02'W | * |  | * | * | * |
| 3366-LJAMM-CNP | Río Negro | 40º47'S | 68º02'W | * |  | * | * | * |
| 3372-LJAMM-CNP | Río Negro | 40º46'S | 68º10'W | * |  | * | * | * |
| 3373-LJAMM-CNP | Río Negro | 40º46'S | 68º10'W | * |  | * | * | * |
| 3386-LJAMM-CNP | Río Negro | 41º30'S | 68º30'W | * |  | * | * | * |
| 3387-LJAMM-CNP | Río Negro | 41º30'S | 68º30'W | * |  | * | * | * |
| 3388-LJAMM-CNP | Río Negro | 41º30'S | 68º30'W | * |  | * | * | * |
| 3389-LJAMM-CNP | Río Negro | 41º30'S | 68º30'W | * |  | * | * | * |
| 3411-LJAMM-CNP | Chubut | 42º24'S | 68º15'W | * |  | * | * | * |
| 3465-LJAMM-CNP | Chubut | 44º09'S | 68º14'W | * |  | * | * | * |
| 3466-LJAMM-CNP | Chubut | 44º09'S | 68º14'W | * |  | * | * | * |
| 3467-LJAMM-CNP | Chubut | 44º09'S | 68º14'W | * |  | * | * | * |
| 3468-LJAMM-CNP | Chubut | 44º09'S | 68º14'W | * |  | * | * | * |
| 3469-LJAMM-CNP | Chubut | 44º09'S | 68º14'W | * |  | * | * | * |
| 3509-LJAMM-CNP | Río Negro | 41º42'S | 70º29'W | * |  | * | * | * |
| 3510-LJAMM-CNP | Río Negro | 41º42'S | 70º29'W | * |  | * | * | * |
| 3511-LJAMM-CNP | Río Negro | 41º42'S | 70º29'W | * |  | * | * | * |
| 3512-LJAMM-CNP | Río Negro | 41º42'S | 70º29'W | * |  | * | * | * |
| 3513-LJAMM-CNP | Río Negro | 41º42'S | 70º29'W | * |  | * | * | * |
| 3519-LJAMM-CNP | Río Negro | 41º45'S | 70º29'W | * |  | * | * | * |
| 3520-LJAMM-CNP | Río Negro | 41º45'S | 70º29'W | * |  | * | * | * |
| 3521-LJAMM-CNP | Río Negro | 41º45'S | 70º29'W | * |  | * | * | * |
| 3522-LJAMM-CNP | Río Negro | 41º45'S | 70º29'W | * |  | * | * | * |
| 3527-LJAMM-CNP | Río Negro | 41º45'S | 70º31'W | * |  | * | * | * |
| 3528-LJAMM-CNP | Río Negro | 41º45'S | 70º31'W | * |  | * | * | * |
| 3762-LJAMM-CNP | Chubut | 44º35'S | 67º53'W | * | * | * | * | * |
| 3763-LJAMM-CNP | Chubut | 44º35'S | 67º53'W | * | * | * | * | * |
| 3764-LJAMM-CNP | Chubut | 44º35'S | 67º53'W | * |  | * | * | * |
| 3765-LJAMM-CNP | Chubut | 44º35'S | 67º53'W | * |  | * | * | * |
| 3766-LJAMM-CNP | Chubut | 44º35'S | 67º53'W | * |  | * | * | * |
| 3772-LJAMM-CNP | Chubut | 44º36'S | 69º08'W | * |  | * | * | * |
| 3803-LJAMM-CNP | Chubut | 45º22'S | 68º34'W | * |  | * | * | * |
| 3804-LJAMM-CNP | Chubut | 45º22'S | 68º34'W | * |  | * | * | * |
| 3814-LJAMM-CNP | Chubut | 42º22'S | 68º10'W | * |  | * | * | * |
| 3815-LJAMM-CNP | Chubut | 42º22'S | 68º10'W | * |  | * | * | * |
| 3816-LJAMM-CNP | Chubut | 42º22'S | 68º10'W | * |  | * | * | * |
| 3817-LJAMM-CNP | Chubut | 42º22'S | 68º10'W | * |  | * | * | * |
| 3818-LJAMM-CNP | Chubut | 42º22'S | 68º10'W | * |  | * | * | * |
| 3819-LJAMM-CNP | Chubut | 42º22'S | 68º10'W | * |  | * | * | * |
| 3820-LJAMM-CNP | Chubut | 42º22'S | 68º10'W | * |  | * | * | * |
| 3821-LJAMM-CNP | Chubut | 42º22'S | 68º10'W | * |  | * | * | * |
| 3822-LJAMM-CNP | Chubut | 42º22'S | 68º10'W | * |  | * | * | * |
| 3884-LJAMM-CNP | Chubut | 43º33'S | 69º04'W | * |  | * | * | * |
| 3885-LJAMM-CNP | Chubut | 42º41'S | 70º02'W | * |  | * | * | * |
| 4451-LJAMM-CNP | Río Negro | 41º10'S | 66º51'W | * | * | * | * | * |
| 4452-LJAMM-CNP | Río Negro | 41º10'S | 66º51'W | * | * | * | * | * |
| 4615-LJAMM-CNP | Chubut | 43º43'S | 67º17'W | * | * | * | * | * |
| 4616-LJAMM-CNP | Chubut | 43º43'S | 67º17'W | * |  | * | * | * |
| 4768-LJAMM-CNP | Chubut | 43º23'S | 69º10'W | * |  | * |  | * |
| 4769-LJAMM-CNP | Río Negro | 40º51'S | 68º10'W | * |  | * | * | * |
| 4770-LJAMM-CNP | Río Negro | 40º51'S | 68º10'W | * |  | * | * | * |
| 4774-LJAMM-CNP | Río Negro | 40º58'S | 66º39'W | * | * | * | * | * |
| 4775-LJAMM-CNP | Río Negro | 40º58'S | 66º39'W | * |  | * | * | * |
| 4776-LJAMM-CNP | Río Negro | 40º58'S | 66º39'W | * |  | * | * | * |
| 4792-LJAMM-CNP | Río Negro | 40º58'S | 66º39'W | * |  | * | * | * |
| 4793-LJAMM-CNP | Río Negro | 40º58'S | 66º39'W | * |  | * | * | * |
| 4794-LJAMM-CNP | Río Negro | 40º58'S | 66º39'W | * |  | * | * | * |
| 4800-LJAMM-CNP | Río Negro | 41º00'S | 67º40'W | * |  | * | * | * |
| 4801-LJAMM-CNP | Río Negro | 41º00'S | 67º40'W | * |  | * | * | * |
| 4802-LJAMM-CNP | Río Negro | 41º00'S | 67º40'W | * |  | * | * | * |
| 4803-LJAMM-CNP | Río Negro | 41º00'S | 67º40'W | * |  | * | * | * |
| 4804-LJAMM-CNP | Río Negro | 41º00'S | 67º40'W | * |  | * | * | * |
| 4806-LJAMM-CNP | Río Negro | 41º00'S | 67º40'W | * |  | * | * | * |
| 4807-LJAMM-CNP | Río Negro | 41º00'S | 67º40'W | * |  | * | * | * |
| 4808-LJAMM-CNP | Río Negro | 40º51'S | 67º40'W | * |  | * | * | * |
| 4809-LJAMM-CNP | Río Negro | 40º51'S | 67º40'W | * | * | * | * | * |
| 4810-LJAMM-CNP | Río Negro | 40º51'S | 67º40'W | * |  | * | * | * |
| 4874-LJAMM-CNP | Chubut | 42º13'S | 66º21'W | * |  | * | * | * |
| 4886-LJAMM-CNP | Río Negro | 40º58'S | 66º39'W | * |  |  |  | * |
| 5437-LJAMM-CNP | Chubut | 42º32'S | 68º01'W | * |  | * | * | * |
| 5438-LJAMM-CNP | Chubut | 42º32'S | 68º01'W | * |  | * | * | * |
| 5439-LJAMM-CNP | Chubut | 42º32'S | 68º01'W | * |  | * | * | * |
| 5481-LJAMM-CNP | Chubut | 42º22'S | 67º34'W | * |  | * | * | * |
| 5482-LJAMM-CNP | Chubut | 42º22'S | 67º34'W | * |  | * | * | * |
| 5483-LJAMM-CNP | Chubut | 42º22'S | 67º34'W | * |  | * | * | * |
| 5484-LJAMM-CNP | Chubut | 42º22'S | 67º34'W | * |  | * | * | * |
| 5485-LJAMM-CNP | Chubut | 42º22'S | 67º34'W | * |  | * | * | * |
| 5504-LJAMM-CNP | Chubut | 42º22'S | 67º35'W | * |  | * | * | * |
| 5513-LJAMM-CNP | Chubut | 42º31'S | 68º01'W | * |  | * | * | * |
| 5514-LJAMM-CNP | Chubut | 42º31'S | 68º01'W | * |  | * | * | * |
| 5515-LJAMM-CNP | Chubut | 42º31'S | 68º01'W | * |  | * | * | * |
| 5516-LJAMM-CNP | Chubut | 42º31'S | 68º01'W | * |  | * | * | * |
| 5517-LJAMM-CNP | Chubut | 42º31'S | 68º01'W | * |  | * | * | * |
| 5518-LJAMM-CNP | Chubut | 42º31'S | 68º01'W | * |  | * | * | * |
| 5519-LJAMM-CNP | Chubut | 42º31'S | 68º01'W | * |  | * | * | * |
| 5583-LJAMM-CNP | Chubut | 42º22'S | 67º34'W | * |  | * | * | * |
| 5594-LJAMM-CNP | Chubut | 42º22'S | 67º24'W | * |  | * | * | * |
| 5595-LJAMM-CNP | Chubut | 42º22'S | 67º24'W | * |  | * | * | * |
| 6023-LJAMM-CNP | Río Negro |  |  |  |  | * | * |  |
| 6028-LJAMM-CNP | Río Negro |  |  |  |  | * | * |  |
| 6029-LJAMM-CNP | Río Negro |  |  |  |  | * | * |  |
| 6030-LJAMM-CNP | Río Negro |  |  |  | * | * | * |  |
| 6031-LJAMM-CNP | Río Negro |  |  |  |  | * | * |  |
| 6039-LJAMM-CNP | Chubut | 42º22'S | 67º30'W | * |  | * | * | * |
| 6040-LJAMM-CNP | Chubut | 42º22'S | 67º30'W | * |  | * | * | * |
| 6043-LJAMM-CNP | Chubut | 42º27'S | 68º46'W | * | * | * | * | * |
| 6044-LJAMM-CNP | Chubut | 42º27'S | 68º46'W | * | * | * | * | * |
| 6070-LJAMM-CNP | Río Negro | 41º55'S | 67º04'W | * |  | * | * | * |
| 6071-LJAMM-CNP | Río Negro | 41º55'S | 67º04'W | * | * | * | * | * |
| 6072-LJAMM-CNP | Chubut | 42º30'S | 67º58'W | * |  | * | * | * |
| 6077-LJAMM-CNP | Chubut | 42º42'S | 68º03'W | * |  | * | * | * |
| 6078-LJAMM-CNP | Chubut | 42º42'S | 68º03'W | * |  | * | * | * |
| 6079-LJAMM-CNP | Chubut | 42º42'S | 68º03'W | * |  | * | * | * |
| 6110-LJAMM-CNP | Chubut | 42º30'S | 67º58'W | * |  | * | * | * |
| 6204-LJAMM-CNP | Chubut | 42º25'S | 68º16'W | * |  | * | * | * |
| 6205-LJAMM-CNP | Chubut | 42º25'S | 68º16'W | * |  | * | * | * |
| 6206-LJAMM-CNP | Chubut | 42º25'S | 68º16'W | * |  | * | * | * |
| 6220-LJAMM-CNP | Río Negro | 41º41'S | 68º04'W | * |  | * | * | * |
| 6221-LJAMM-CNP | Río Negro | 41º41'S | 68º04'W | * |  | * | * | * |
| 6222-LJAMM-CNP | Río Negro | 41º41'S | 68º04'W | * |  | * | * | * |
| 6223-LJAMM-CNP | Río Negro | 41º41'S | 68º04'W | * |  | * | * | * |
| 6238-LJAMM-CNP | Río Negro | 41º30'S | 68º35'W | * |  | * | * | * |
| 6239-LJAMM-CNP | Río Negro | 41º30'S | 68º35'W | * |  | * | * | * |
| 6240-LJAMM-CNP | Río Negro | 41º30'S | 68º35'W | * |  | * | * | * |
| 6241-LJAMM-CNP | Río Negro | 41º30'S | 68º35'W | * | * | * | * | * |
| 6737-LJAMM-CNP | Chubut | 42°21'S | 67°27'W | * | * |  |  | * |
| 6756-LJAMM-CNP | Chubut | 42°25'S | 68°17'W | * | * |  |  | * |
| 6779-LJAMM-CNP | Río Negro | 41°23'S | 66°57'W | * |  |  |  | * |
| 6780-LJAMM-CNP | Río Negro | 41°23'S | 66°57'W | * |  |  |  | * |
| 6781-LJAMM-CNP | Río Negro | 41°23'S | 66°57'W | * |  |  |  | * |
| 6782-LJAMM-CNP | Río Negro | 41°23'S | 66°57'W | * |  |  |  | * |
| 6783-LJAMM-CNP | Río Negro | 41°23'S | 66°57'W | * |  |  |  | * |
| 6784-LJAMM-CNP | Río Negro | 41°23'S | 66°57'W | * |  |  |  | * |
| 6785-LJAMM-CNP | Río Negro | 41°23'S | 66°57'W | * |  |  |  | * |
| 6786-LJAMM-CNP | Río Negro | 41°23'S | 66°57'W | * |  |  |  | * |
| 6787-LJAMM-CNP | Río Negro | 41°23'S | 66°57'W | * |  |  |  | * |
| 6788-LJAMM-CNP | Río Negro | 41°23'S | 66°57'W | * |  |  |  | * |
| 6789-LJAMM-CNP | Río Negro | 41°23'S | 66°57'W | * |  |  |  | * |
| 6790-LJAMM-CNP | Río Negro | 41°23'S | 66°57'W | * |  |  |  | * |
| 6791-LJAMM-CNP | Río Negro | 41°23'S | 66°57'W | * |  |  |  | * |
| 6814-LJAMM-CNP | Río Negro | 40°46'S | 68°11'W | * |  |  |  | * |
| 6815-LJAMM-CNP | Río Negro | 40°46'S | 68°11'W | * | * |  |  | * |
| 6816-LJAMM-CNP | Río Negro | 40°46'S | 68°11'W | * |  |  |  | * |
| 6817-LJAMM-CNP | Río Negro | 40°46'S | 68°11'W | * |  |  |  | * |
| 6818-LJAMM-CNP | Río Negro | 40°46'S | 68°11'W | * |  |  |  | * |
| 6836-LJAMM-CNP | Río Negro | 41°23'S | 66°57'W | * |  |  |  | * |
| 6848-LJAMM-CNP | Río Negro | 40°46'S | 68°11'W | * |  |  |  | * |
| 6898-LJAMM-CNP | Chubut | 42°25'S | 68°17'W | * | * |  |  | * |
| 6899-LJAMM-CNP | Chubut | 42°25'S | 68°17'W | * | * |  |  | * |
| 6900-LJAMM-CNP | Chubut | 42°25'S | 68°17'W | * |  |  |  | * |
| 6902-LJAMM-CNP | Chubut | 42°25'S | 68°17'W | * |  |  |  | * |
| 6953-LJAMM-CNP | Chubut | 42°32'S | 68°01'W | * |  |  |  | * |
| 6954-LJAMM-CNP | Chubut | 42°32'S | 68°01'W | * |  |  |  | * |
| 6955-LJAMM-CNP | Chubut | 42°32'S | 68°01'W | * |  |  |  | * |
| 6956-LJAMM-CNP | Chubut | 42°32'S | 68°01'W | * |  |  |  | * |
| 6957-LJAMM-CNP | Chubut | 42°32'S | 68°01'W | * |  |  |  | * |
| 6958-LJAMM-CNP | Chubut | 42°32'S | 68°01'W | * |  |  |  | * |
| 6959-LJAMM-CNP | Chubut | 42°3'S | 68°01'W | * |  |  |  | * |
| 6982-LJAMM-CNP | Río Negro | 39°44'S | 68°29'W | * |  |  |  | * |
| 6983-LJAMM-CNP | Río Negro | 39°44'S | 68°29'W | * | * |  |  | * |
| 6984-LJAMM-CNP | Río Negro | 39°44'S | 68°29'W | * | * |  |  | * |
| 6985-LJAMM-CNP | Río Negro | 39°44'S | 68°29'W | * |  |  |  | * |
| 6986-LJAMM-CNP | Río Negro | 39°44'S | 68°29'W | * |  |  |  | * |
| 6987-LJAMM-CNP | Río Negro | 39°44'S | 68°29'W | * |  |  |  | * |
| 6988-LJAMM-CNP | Río Negro | 39°44'S | 68°29'W | * |  |  |  | * |
| 6989-LJAMM-CNP | Río Negro | 39°44'S | 68°29'W | * | * |  |  | * |
| 6990-LJAMM-CNP | Río Negro | 39°44'S | 68°29'W | * | * |  |  | * |
| 6991-LJAMM-CNP | Río Negro | 39°44'S | 68°29'W | * |  |  |  | * |
| 6992-LJAMM-CNP | Río Negro | 39°44'S | 68°29'W | * |  |  |  | * |
| 6993-LJAMM-CNP | Río Negro | 39°44'S | 68°29'W | * |  |  |  | * |
| 6994-LJAMM-CNP | Río Negro | 39°44'S | 68°29'W | * |  |  |  | * |
| 6996-LJAMM-CNP | Río Negro | 40°18'S | 69°22'W |  | * |  |  |  |
| 7076-LJAMM-CNP | Chubut | 42°02'S | 70°18'W | * | * |  |  | * |
| 7077-LJAMM-CNP | Chubut | 42°02'S | 70°18'W | * | * |  |  | * |
| 7078-LJAMM-CNP | Chubut | 42°02'S | 70°18'W | * |  |  |  | * |
| 7079-LJAMM-CNP | Chubut | 42°02'S | 70°18'W | * |  |  |  | * |
| 7080-LJAMM-CNP | Chubut | 42°02'S | 70°18'W | * |  |  |  | * |
| 7102-LJAMM-CNP | Chubut | 42°32'S | 68°01'W |  |  |  |  |  |
| 7517-LJAMM-CNP | Río Negro | 40º58'S | 66º39'W |  |  |  |  |  |
| 7835-LJAMM-CNP | Chubut | 42°25'S | 68º14'W | * |  |  |  | * |
| 7839-LJAMM-CNP | Chubut | 42°25'S | 68º14'W | * |  |  |  | * |
| 8135-LJAMM-CNP | Chubut | 42°30'S | 67°21'W |  |  |  |  |  |
| 8185-LJAMM-CNP | Chubut | 42°41'S | 68º13'W | * |  |  |  | * |
| 8215-LJAMM-CNP | Río Negro | 41°17'S | 66º28'W | * |  |  |  | * |
| 8216-LJAMM-CNP | Río Negro | 41°17'S | 66º28'W | * |  |  |  | * |
| 8217-LJAMM-CNP | Río Negro | 41°17'S | 66º28'W | * |  |  |  | * |
| 8452-LJAMM-CNP | Río Negro | 41°17'S | 66º28'W | * |  |  |  | * |
| 8453-LJAMM-CNP | Río Negro | 41°17'S | 66º28'W | * |  |  |  | * |
| 8818-LJAMM-CNP | Chubut | 43º23'S | 69º10'W | * | * |  |  | * |
| 8819-LJAMM-CNP | Chubut | 43º23'S | 69º10'W | * | * |  |  | * |
| 8820-LJAMM-CNP | Chubut | 43º23'S | 69º10'W | * |  |  |  | * |
| 8821-LJAMM-CNP | Chubut | 43º23'S | 69º10'W | * |  |  |  | * |
| 8823-LJAMM-CNP | Chubut | 42º56'S | 68º31'W | * |  |  |  | * |
| 8824-LJAMM-CNP | Chubut | 42º56'S | 68º31'W | * |  |  |  | * |
| 8857-LJAMM-CNP | Chubut | 42º39'S | 70º22'W | * |  |  |  | * |
| 8858-LJAMM-CNP | Chubut | 42º39'S | 70º22'W | * | * |  |  | * |
| 8859-LJAMM-CNP | Chubut | 42º39'S | 70º22'W | * |  |  |  | * |
| 8860-LJAMM-CNP | Chubut | 42º39'S | 70º22'W | * |  |  |  | * |
| 8861-LJAMM-CNP | Chubut | 42º39'S | 70º22'W | * |  |  |  | * |
| 8862-LJAMM-CNP | Chubut | 42º39'S | 70º22'W | * |  |  |  | * |
| 8863-LJAMM-CNP | Chubut | 42º39'S | 70º22'W | * |  |  |  | * |
| 8864-LJAMM-CNP | Chubut | 42º39'S | 70º22'W | * |  |  |  | * |
| 8865-LJAMM-CNP | Chubut | 42º39'S | 70º22'W | * |  |  |  | * |
| 8866-LJAMM-CNP | Chubut | 42º39'S | 70º22'W | * |  |  |  | * |
| 8867-LJAMM-CNP | Chubut | 42º39'S | 70º22'W | * |  |  |  | * |
| 8868-LJAMM-CNP | Chubut | 42º41'S | 70º22'W | * |  |  |  | * |
| 8869-LJAMM-CNP | Chubut | 42º41'S | 70º22'W | * |  |  |  | * |
| 8870-LJAMM-CNP | Chubut | 42º41'S | 70º22'W | * |  |  |  | * |
| 10955-LJAMM-CNP | Río Negro | 41º45'S | 67º59'W |  | * |  |  |  |
| 10956-LJAMM-CNP | Río Negro | 41º45'S | 67º59'W |  |  |  |  |  |
| 11123-LJAMM-CNP | Río Negro | 41º05'S | 67º53'W |  | * |  |  |  |
| 11133-LJAMM-CNP | Chubut | 42º 31'S | 68º 17'W |  |  |  |  |  |
| 11134-LJAMM-CNP | Chubut | 42º 31'S | 68º 17'W |  |  |  |  |  |
| 11135-LJAMM-CNP | Chubut | 42º 31'S | 68º 17'W |  | * |  |  |  |
| 11141-LJAMM-CNP | Chubut | 42º 31'S | 68º 17'W |  |  |  |  |  |
| 11147-LJAMM-CNP | Chubut | 42º 31'S | 68º 17'W |  |  |  |  |  |
