# Supplementary material for: Early stages of divergence: phylogeography, climate modeling, and morphological differentiation in the South American lizard Liolaemus petrophilus (Squamata: Liolaemidae)
Source: Ecol Evol. 2012 Apr;2(4):792–808. doi: 10.1002/ece3.78 (PMC3399201; doi:10.1002/ece3.78)
Supplement: Supplementary file 3 [file ece30002-0792-SD3.doc]

Supplementary Table 2 Continuous and meristic characters used in the classic morphometrics analysis of *Liolaemus petrophilus*. Characters that were significantly different (P>0.05) between clades (North vs South) and between sexes within clades are marked with an asterisk. Characters showing sexual dimorphism within either the North or South clades were not tested for differences between haploclades (marked with Z).

| Variable | North/South (df 166) | North (df 94) | South (df 70) |
| --- | --- | --- | --- |
| Snout-vent length (SVL) | Z | * |  |
| Head length (HL) | Z | * | * |
| Head width (HW) | Z | * |  |
| Head height (HH) | Z | * |  |
| Distance between nares (DBN) | Z | * |  |
| Axial-Groin distance (AGD) | * |  |  |
| Front limb length (FLL) | Z | * |  |
| Cubitus-radium length (CRL) | Z | * | * |
| Hind limb length (HLL) | Z | * | * |
| Scales around body (SAB) |  |  |  |
| Third finger lamellae of front limb (TFL) | * |  |  |
| Fourth toe lamella of hind limb (FTL) | * |  |  |
